# Lipid Thermal Fingerprints of Long-term Stored Seeds of Brassicaceae

**DOI:** 10.3390/plants8100414

**Published:** 2019-10-14

**Authors:** Sara Mira, Jayanthi Nadarajan, Udayangani Liu, Maria Elena González-Benito, Hugh W. Pritchard

**Affiliations:** 1 Departamento de Biotecnología-Biología Vegetal, E.T.S.I. Agronómica, Alimentaria y de Biosistemas. Universidad Politécnica de Madrid, 28040 Madrid, Spain; sara.mira@upm.es (S.M.); me.gonzalezbenito@upm.es (M.E.G.-B.); 2 Royal Botanic Gardens Kew, Wellcome Trust Millennium Building, Wakehurst, Ardingly, West Sussex RH17 6TN, UK; u.liu@kew.org (U.L.); h.pritchard@kew.org (H.W.P.); 3 The New Zealand Institute for Plant and Food Research Limited, Private Bag 11600, Palmerston North 4442, New Zealand

**Keywords:** crop wild relatives, longevity, differential scanning calorimetry (DSC), seed banking, conservation

## Abstract

Thermal fingerprints for seeds of 20 crop wild relatives of Brassicaceae stored for 8 to 44 years at the Plant Germplasm Bank—Universidad Politécnica de Madrid and the Royal Botanic Gardens, Kew’s Millennium Seed Bank—were generated using differential scanning calorimetry (DSC) and analyzed in relation to storage stability. Relatively poor storing oily seeds at −20 °C tended to have lipids with crystallization and melting transitions spread over a wide temperature range (c. 40 °C) that spanned the storage temperature, plus a melting end temperature of around 15 °C. We postulated that in dry storage, the variable longevity in Brassicaceae seeds could be associated with the presence of a metastable lipid phase at the temperature at which they are being stored. Consistent with that, when high-quality seed samples of various species were assessed after banking at −5 to −10 °C for c. 40 years, melting end temperatures were observed to be much lower (c. 0 to −30 °C) and multiple lipid phases did not occur at the storage temperature. We conclude that multiple features of the seed lipid thermal fingerprint could be used as biophysical markers to predict potential poor performance of oily seeds during long-term, decadal storage.

## 1. Introduction

Optimal conditions of humidity and temperature for the long-term storage of seeds are still the subject of debate, principally as there are few long-term data sets. Seeds that tolerate further drying may benefit with improved longevity, although the minimum safe humidity for long-term storage appears to vary with species, and possibly with seed lot. For orthodox seeds, dehydration to c. 11% equilibrium relative humidity (equivalent to about 2.5–6.5% moisture content, depending on seed oil content) and subsequent storage at −20 °C has been shown to be safe [1]. Further improved longevity may or may not be achieved by drying below these moisture levels, into the so-called ultra-dry zone; improvement could facilitate storage for many years without the need for cooling [2,3,4].

Moreover, seeds with partial desiccation tolerance to c. 10–15% moisture content (c. 50–80% relative humidity, RH) may not store well at −20 °C. These latter seeds, oft-called “intermediate”, tend to lose germinability quite rapidly at cool (5 °C) or cold (−20 °C) storage temperatures, for example, within 1 year [5]. However, it is possible that such seeds could initially respond to drying as if an orthodox seed (i.e., fully desiccation tolerant), but that the cold sensitivity plays out over the longer term. In this case, perhaps intermediate seeds are a subset of orthodox seeds with short lifespans. Evidence of variability in the level of partial desiccation tolerance, time and temperature dependency of the cold sensitivity response, suggests a number of dry seed syndromes or types [6]. Consequently, this category of seeds (orthodox intermediates; intermediate orthodoxy?) is still difficult to determine, and would benefit from the combining of the physiological response with an understanding of mechanisms.

Recently, Walters [7] proposed three means of identifying “intermediate” seeds: (1) seeds that tolerate desiccation between orthodox and recalcitrant seeds; (2) seeds that show inconsistent storability to temperatures between +10 and −30 °C; and (3) seeds with short lifespan regardless of their drying and cooling methods. Interestingly, these types of behaviors are apparent mostly in oily seeds of a number of tropical, subtropical, and warm temperate species (e.g., citrus [8,9], coffee [10], *Cuphea* [11], neem [12]; orchids [13], papaya [14], and tea [15]). Being able to rapidly diagnose such seeds would have practical value and open up the possibility of wide-scale screening of germplasm held in the world’s genebanks.

Seed lipid content has long been thought to be a determinant of seed ageing (reviewed by Priestley, [16]), with longevity and lipid content possibly being inversely related. However, analysis of numerous datasets has shown that lipid content is not, in itself, a reliable predictor of seed lifespan [17,18,19,20,21]. Lipid composition correlates better with seed survival as a result of differing susceptibility to oxidation [22,23] or through effects on thermal behavior. For example, the energy associated with lipid melting has been used as a descriptor of viability loss [24,25]. Also, storing oily seeds at cold temperatures, at which lipids may exist in multiple physical states can compromise seed lifespan [11,13,26], with the need to melt lipid crystals impacting on optimal conditions for subsequent germination [11,26]. Overall, it seems that oily seeds with lipids that complete melting between about 5 °C and 25 °C appear more sensitive to dry, cold storage at c. 5 to −30 °C; for example *Azadirachta indica* [12], *Cattleya aurantiaca* [13], *Cuphea parsonia, Cuphea carthagenensis, Cuphea palustris* and *Cuphea glossostoma* [26], and *Carica papaya* (Pritchard HW, pers comm). Moreover, poorer cryopreservation success (i.e., fewer normal seedlings) in *Citrus garrawayi* seed stored at 3% moisture content has been correlated with the presence of higher temperature melting lipids at c. 11 °C [8].

Thermal analysis may be used to characterize the physical properties of biological samples and may have potential as a rapid, noninvasive tool to understand the variability in storage performance in relation to features of the seed lipids, namely: lipid quantity, lipid composition, and physical properties such crystallization and melting [11,13,26].

To assess the potential of thermal analysis to predict oily seed storage stability, we selected mainly underutilized crop wild relatives (CWR) of Brassicaceae. CWR are wild plants genetically related to crops of socioeconomic importance that have not passed through the genetic selection and manipulation of domestication [27,28]. There is ongoing interest in wild Brassicaceae germplasm to be used as the primary gene pool for vegetable crops, such as cauliflower and broccoli [29,30,31]. Genera within Brassicaceae have a wide distribution, the seeds have a high proportion of oil [32], and there is significant interspecies variation in seed longevity [21,33]. The Plant Germplasm Bank—Universidad Politécnica de Madrid (UPM) holds a historical collection of approximately 750 taxa of the Brassicaceae family. The collection was designated as the base bank for Brassicaceae wild members by the International Plant Genetic Resources Institute (now Bioversity International) in 1983 and has reported high viability of ultra-dried seeds, stored for up to 40 years at high subzero temperatures (between −5 and −10 °C) [2,3,4]. The Millennium Seed Bank of the Royal Botanic Gardens Kew (RBG Kew) holds, at −20 °C, seed lots of over 680 species from the Brassicaceae family. The storage performance of Brassicaceae seeds varies between species and with storage procedures [2,3,4,20,21,34]. Thus, an analysis of seed survival of Brassicaceae species in these two globally significant genetic resource collections offers a unique opportunity to assess interspecies differences in post-storage germinability.

The hypothesis tested is that features of the lipid thermal fingerprint might be used as a means of rapidly identifying potentially at-risk accessions of oilseeds under cold storage, based on the performance of seed lots of Brassicaceae species stored for up to 44 years.

## 2. Results

### 2.1. Seed Germination

Five species (*Conringia orientalis*, *Descurainia sophia*, *Moricandia arvensis*, *Sinapis arvensis,* and *Sisymbrium orientale*) showed very low or no germination at the beginning of storage, possibly due to dormancy, however germination improved significantly to 70–100% at the end of the storage (Table 1). *Arabis turrita* stored at UPM was an exception, having an initial germination of 0% and just 4% germination after storage. *Arabis turrita* seeds were dormant during collection and maintained this dormancy throughout storage: a dormancy-breaking treatment (48 h soak in 1 g/L gibberellic acid GA_3_) had limited success. In contrast, seeds of two species from RBG Kew, *Alliaria petiolata* and *Lesquerella gordonii* showed a drastic decline in germination following storage for 32 and 8 years from c. 90% to 24% and 0% respectively, without any concerns regarding the germination test (Table 1).

When seeds were sown for germination after DSC thermal analysis, most of the species retained high germination; although two species had significantly higher (*Arabis turrita, Coincya rupestris*) and one species (*Matthiola sinuata*) had significantly lower germination after cooling and warming cycles (Table 1).

### 2.2. Lipid Thermal Fingerprints, Lipid Content, and Fatty Acid Compositions

Based on published work, seed lipid content among the studied species varied from 13% (*Alyssoides utriculata*) to 40% (*Brassica napus*); the average lipid content for all species studied was 26% (Table 2).

**Figure 1:**
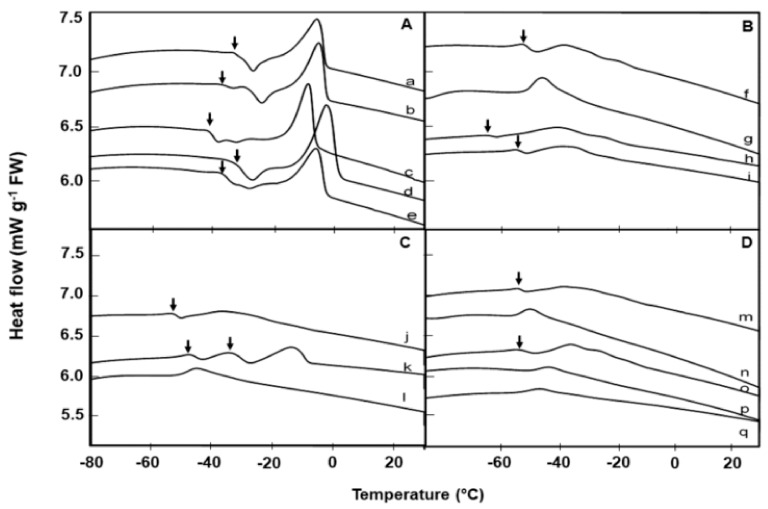
Comparative warming thermograms (at 10 °C min^−1^) of lipid thermal transition events in seeds of 17 Brassicaceae species from UPM collection, measured using differential scanning calorimetry. Lipid crystallization (↓) events during warming, prior to the main melting transitions are evident in 12 species. **A:** Species with high lipid melt enthalpy: *Barbarea intermedia* (a), *Erucastrum abyssinicum* (b), *Conringia orientalis* (c), *Brassica napus* (d), and *Sinapis arvensis* (e). **B:** Species with medium lipid melt enthalpy: *Coincya rupestris* (f), *Matthiola sinuata* (g), *Descurainia sophia* (h), *Erysimum odoratum* (i). **C:** Species with medium lipid melt enthalpy: *Erysimum repandum* (j), *Erysimum cheiri* (k), *Alyssum saxatile* (l). **D:** Species with low lipid melt enthalpy: *Sisymbrium orientale* (m), *Arabis turrita* (n), *Moricandia arvensis* (o), *Matthiola incana* (p), *Alyssoides utriculata* (q).

**Figure 2:**
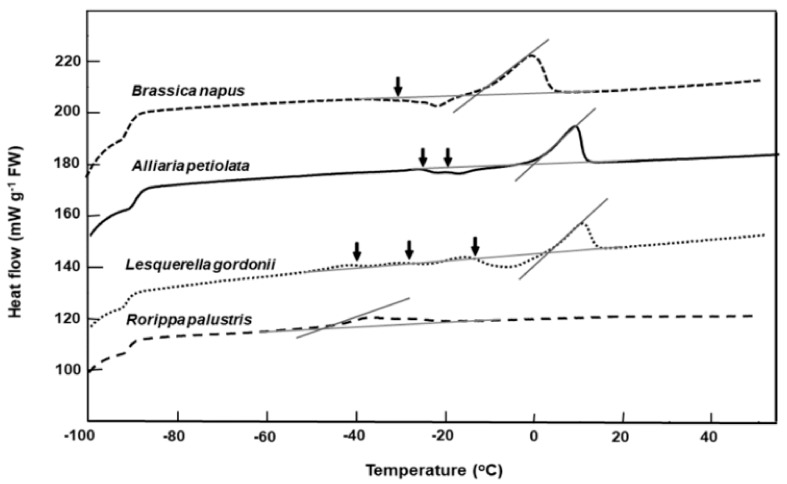
Comparative warming thermograms for seeds of four Brassicaceae species from the RBG Kew collection. Seeds were cooled/warmed at ±10 °C min^−1^. Onset temperatures for the main lipid melts coincide with the intersect between the tangent to the steepest part of the melting peak and the scanning baseline. Lipid crystallization during warming is indicated (↓). Multiple crystallization events around the storage temperature (−20 °C) are evident for *Alliaria petiolata* and *Lesquerella gordonii.*

Dry seeds of all 20 species exhibited lipid transitions during cooling/warming runs in the DSC. Details of the rewarming thermograms are summarized in Table 2. *Alliaria petiolata*, *Barbarea intermedia, Brassica napus*, *Conringia orientalis, Erucastrum abyssinicum*, *Lesquerella gordonii,* and *Sinapis arvensis* had higher onset and end lipid melt temperatures (Figure 1 and Figure 2, Table 2). The prominent fatty acids for each species are also summarized in Table 2, based on published data. In relation to fatty acid composition, eight species (*Alliaria petiolata, Brassica napus, Coincya rupestris*, *Conringia orientalis*, *Erucastrum abyssinicum*, *Erysimum odoratum*, *Lesquerella gordonii, and Sinapis arvensis*) have 25% or more long carbon chains (>20 carbon) and, apart from *Coincya rupestris* and *Erysimum odoratum*, have high main lipid melting points ranging from −8 to 13 °C (Table 2).

The number of melting peaks, peak size, and temperature of melting transition varied among species. For example, *Brassica napus,* which had the highest lipid content also showed the highest lipid melt enthalpy based on lipid content, followed by *Barbarea intermedia* and *Alliaria petiolata* (Table 2). In Figure 1, in accordance with their lipid melt enthalpy (ΔH), the 17 species from the UPM collection were grouped into three categories, with melt enthalpies that are high (≥19 mJ g^−1^ dry weight, DW; Figure 1A), medium (>5.9 and <19 mJ g^−1^ DW; Figure 1B, C) or low (<4.4 mJ g^−1^ DW; Figure 1D).

Warming thermograms for seed of *Alliaria petiolata, Brassica napus, Lesquerella gordonii,* and *Rorippa palustris* that had been long-term stored in the RBG Kew bank are shown in Figure 2. *Brassica napus* and *Rorippa palustris* seeds had good long-term survival, with high germination after 33 and 11 years of storage (Table 1) and with lipid melt end temperatures of 2 °C and −38 °C, respectively (Figure 2). In contrast, the poorer-storing *Alliaria petiolata* and *Lesquerella gordonii* exhibited thermograms with two or three lipid crystallization/partial melting transitions that spanned the storage temperature of −20 °C (Figure 2). Seeds of these species also had higher lipid melt end temperatures of c. 17 °C (Figure 2).

The onset temperatures for the lipid melt peak varied among species; being lowest (c. −51 °C) for *Alyssum saxatile* and highest (c. 2 °C) in *Alliaria petiolata* (Table 2). *Erysimum cheiri* showed a distinct, double lipid crystallization transition during the heating phase between −30 and −50 °C (Figure 1C), far below the storage temperature. 

**Figure 3:**
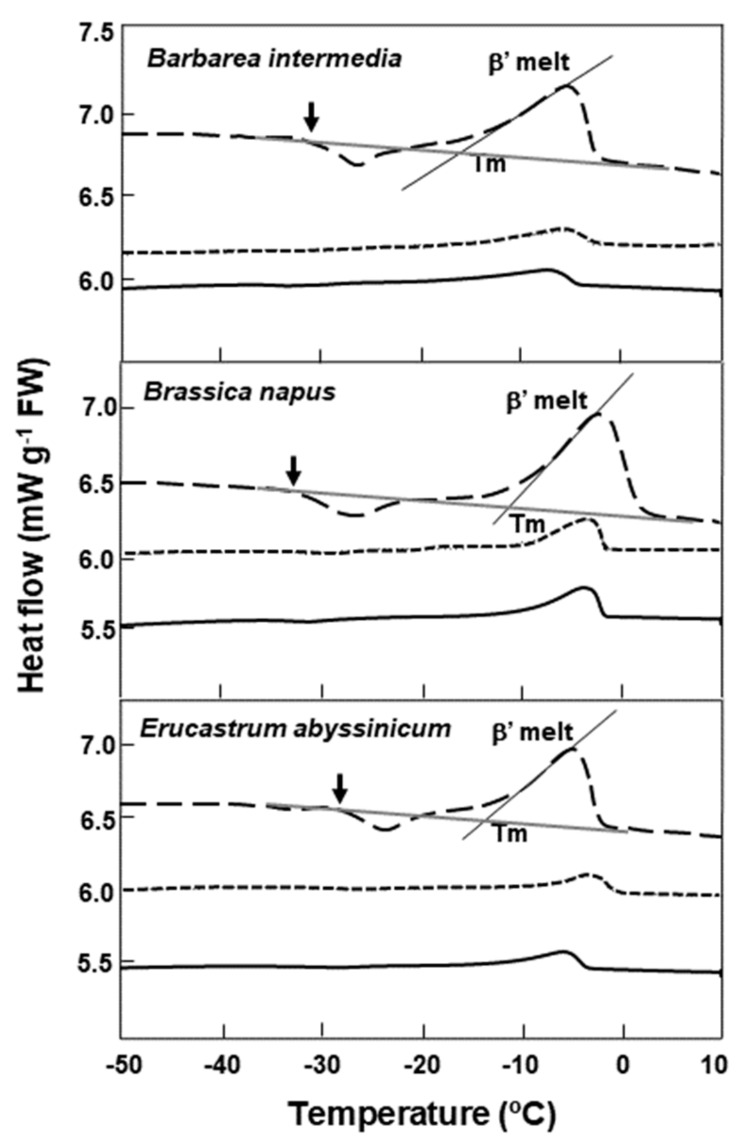
DSC comparative warming thermograms for seeds of three Brassicaceae species at various cooling/warming rates. Three warming rate treatments are shown: ±10 °C min^−1^ (dashed line), showing the onset of crystallization (↓) and melting (β’) of the lipid during warming; ±2 °C min^−1^ (solid line); and ±2 °C min^−1^ with samples annealed for 1 h at −5 °C during cooling (short dashed line). Onset temperatures for the main lipid melts (Tm) coincide with the intersect between the tangent to the steepest part of the melting peak and the scanning baseline; and are shown only for the ±10 °C min^−1^ temperature regime. The loss of lipid crystallization events during warming are evident in the slower-cooled/warmed and annealed samples.

For *Barbarea intermedia, Brassica napus,* and *Erucastrum abyssinicum*, when the cooling rate in the DSC was reduced to 2 °C min^−1^, the lipid melt end temperature decreased slightly, for example, from 1.7 to −1.7 °C for *Brassica napus* (Figure 3). The use of a slower cooling/warming rate and annealing at −5 °C for 1 h during cooling had little effect on the lipid melt onset temperatures, which spanned c. 3 °C for all three species. However, the loss of lipid crystallization events during warming were noted in the slower cooled/warmed samples (Figure 3). 

## 3. Discussion

In this study, we attempted to establish a relationship between lipid thermal fingerprints and seed survival over the longer term (i.e., decades). Lipid phase transitions were detected in DSC scans of dry seeds of all species, and the scans were consistent with published evidence on Brassicaceae species generally producing oily seeds. Vertucci [24] measured changes in the physical properties of lipids in dry cool-stored seeds with similar genetic constituents, harvested at different times (11–31 years) using DSC and found that in most cases, the qualitative differences in the thermograms for fresh and deteriorated seeds were reflected in differences in the temperature and/or the energy of the lipid transition. Changes in the glass-forming tendencies among fresh and older seeds were noted in onion, lettuce, sunflower, corn, watermelon, and pepper [24]. These findings suggest that storing seed for extended periods of time at specific temperatures could affect the lipid phase “behavior” (melt onset and end temperatures, enthalpies, glass transitions, and crystallization events). We were unable to implement such direct comparisons between fresh and stored seed in this instance. Whilst germination assessments were made before or soon after placing seeds in the storage environment, no thermal analyses were undertaken on the seed lots when they were placed into storage 40 years ago. Notwithstanding, it is still valuable to understand if there are any thermal features of seeds that can be used to differentiate storage stability in historical collections.

In the present study we demonstrated that Brassicaceae seeds from the UPM and RBG Kew collections have highly variable thermal properties. Moreover, high variability in longevity has been reported among species within the Brassicaceae family, and even within genera, during accelerated aging conditions [33] and in cold storage at RBG Kew based on our study, also Probert et al. [20], and elsewhere, for example, the National Centre for Genetic Resources Preservation, USA [21]. For example, at RBG Kew, only about 70% of the Brassicaceae accessions tested after 20 years of storage at −20 °C and 15% RH showed no viability loss [20]. Walters et al. [21] partitioned Brassicaceae species seed longevity into either short or long shelf lives, with no species with medium longevities.

Germination test results of 12 species from the RBG Kew stored at −20 °C showed that two species (*Alliaria petiolata* and *Lesquerella gordonii*) are relatively short-lived (i.e., unexpectedly lose some viability within 20 years). Interestingly, seeds of both of these species have lipids with higher end melt temperatures (at around 17 °C). This feature of the thermal fingerprint in poor-storing seeds is in general agreement with the high melting temperatures reported in other poor-storing oilseeds of *Cuphea* [26] and citrus [8]. The decline in viability for the two species in this study (*Alliaria petiolata* and *Lesquerella gordonii*) that have a lipid main melting temperature midpoint at around 12 °C could be one indicator for putative short life in oilseeds in conventional seed bank storage.

In addition to the effects of specific cold temperatures, cooling rates in dry seeds also can influence lipid’s polymorphic or metastable crystal formation, the effects of which could be diminished by slow cooling or annealing [38]. In agreement with this, we note that seeds of *Barbarea intermedia, Brassica napus,* and *Erucastrum abyssinicum* subjected to a lower cooling rate of 2 °C min^−1^, lost crystallization events around −25 °C during warming, indicative of the loss of metastable crystals related to lipids. This is also true for samples slow-cooled (2 °C min^−1^) and annealed at −5 °C for 1 h. It is known that storage lipids in seeds in the form of triacylglycerol can crystallize into several polymorphic forms, designated as α, β, and β^1^ in increasing order of melting point, packing density, and thermodynamic stability, during cooling at a rate of 10 °C min^−1^ [39]. The α-form is a metastable crystal which can undergo a polymorphic transition to form a more stable crystal [40]. Subtle changes in these metastable lipids could alter the cis−trans conformation of their fatty acids and interfere with the lipid−lipid and lipid−nonlipid moiety interactions [21,26,41]. The mixed states of the lipids and the overlapping of crystalization/onset melts are postulated to have a detrimental effect on the storability of seeds at specific low temperatures. The two poor storers at RBG Kew (*Alliaria petiolata* and *Lesquerella gordonii*) support this suggestion.

Characterization of lipid phase thermal transitions in dry seeds with altered germination rates, imbibitional damage, and decreased seed quality post-storage have been reported before using DSC [21,24,26,42]. We extended this reasoning to UPM seeds to predict that the quality of seeds of species for which lipids are mostly fluid at the storage temperature (c. −5 °C) are less likely to be compromised than seeds of those species with a lipid melt end temperature above −5 °C. In this regard, and based on the thermograms, storage of four species (*Barbarea intermedia, Brassica napus, Erucastrum abyssinicum,* and *Sinapis arvensis)* at c. −5 °C might be considered to be risky. For *Erucastrum abyssinicum*, which had a melting point of −4.7 °C, this seems to be the case, as the seeds showed a significant reduction in germination from 100% to 90% after 44 year storage at UPM. More interestingly, *Sinapis arvensis,* which had a lipid melt point at −6.3 °C also showed a significant decline in germination to 73% after 43 years at UPM and 90% after 12 years storage at RBG Kew, respectively. The association between physical changes in the seeds with reducing seed quality can, therefore, be realized over the long term, unlike the often shorter-term effects observed in intermediate seeds.

Due to the known interspecies variability in seed longevity, it is not surprising that we found that not all Brassicaceae species’ seeds tolerate dry and cold storage for four decades. In decade-long stored fern spores, the complexity, temperature, and total enthalpy of lipid transitions has been observed to vary as a result of changes in molecular mobility in the glassy state and the seeds thermal history [43]. Interestingly, the UPM seed bank stored seeds have had very different thermal history compared with RBG Kew stored seeds. The UPM seeds were stored at −5 °C for 15 years (from 1966 to 1981), at −10 °C for 23 years (until 2004), and at −5 °C thereafter; whilst the RBG Kew seeds were stored at −20 °C continually. Only *Brassica napus* seeds, albeit of different accessions, were stored in both seed banks, permitting a direct comparison of their thermal properties. In agreement with Ballesteros et al. [43], we note that the melt onset, melt end, and melt peak temperatures are slightly lower for RBG Kew stored seeds compared with UPM stored seeds (temperatures in brackets in Table 2). This suggests that the molecular mobility, chemical reactivity, and molecular stability for these two seed populations is very different at the two different storage temperatures (−5 vs. −20 °C). A main peak at lower temperatures in the seeds stored at −20 °C compared with −5 °C could indicate that the molecular structure of crystals is constantly reorganizing into denser and lower energy forms and global motion is more restricted at −20 °C stored seeds, which would be reflected in the lower ageing kinetics [44]. Hence, it could be predicted that *Brassica napus* seeds at RBG Kew would store longer compared with UPM stored seeds, consistent also with the known benefits to longevity of lowering storage temperature for orthodox seeds [45].

*Arabis turrita* seeds were dormant during collection and maintained this dormancy throughout the 43 years of storage, with dormancy-breaking treatment (48 h soaking in 1 g/L GA_3_) being applied with little success. In contrast, *Conringia orientalis, Descurainia sophia, Moricandia arvensis, Sinapis arvensis,* and *Sisymbrium orientale* seeds were reported to show significant dormancy loss while in storage, as discussed in Perez-Garcia et al. [4]. It is interesting to note that most species’ seeds can germinate after a cooling and warming cycle (room temperature to −100 °C, and then warmed to 30 °C) in the DSC. In the case of *Arabis turrita* seed, germination increased considerably (4% to 24%) after thermal treatment, similar to preheating cold-stored *Cuphea* seed to 45 °C prior to the onset of the germination test [46]. In this regard, it is clear that cooling and reheating can influence the germination ability of seeds of some species, possibly as a result of melting residual components of crystallized lipids.

Lipid composition has been reported to correlate with seed survival as a result of differing susceptibility to oxidation [22,23,47] or through effects on thermal behavior. In this study, we note that eight species (*Alliaria petiolata, Brassica napus, Coincya rupestris*, *Conringia orientalis*, *Erucastrum abyssinicum*, *Erysimum odoratum*, *Lesquerella gordonii,* and *Sinapis arvensis*) have 25% or more of their fatty acid composition as long carbon chains (>20 carbon). Apart from *C. rupestris* and *E. odoratum*, the other species have high lipid melting points ranging from −8 to 13 °C (Table 2). Interestingly, seeds of four of these species (*Alliaria petiolata, Erucastrum abyssinicum*, *Lesquerella gordonii,* and *Sinapis arvensis*) show declines in germination during storage in both seed banks (Table 1). On the contrary, both *Conringia orientalis* and *Brassica napus* species seeds have long-carbon-chain fatty acids and retain higher germination over the long term. As seeds of *Brassica napus* have also been reported as short-lived [17], accessions of this species are candidates for studies on relations between storability and fatty acid composition, chain length, oil body stability, and thermal properties.

## 4. Materials and Methods

### 4.1. Plant Material

For Universidad Politécnica de Madrid (UPM) seed collection, experiments were performed on seeds of 17 Brassicaceae species (Table 1), stored in ultra-dry, cold conditions. Accessions had been placed into storage between 1966 and 1967 inside flame-sealed glass vials containing approximately 39 to 104 mg of dry seeds, together with silica gel, separated by a filter paper divider, following the method described by Gómez-Campo [35] (Figure S1). Vials were stored at −5 °C from 1966 to 1981, at −10 °C until 2004, and at −5 °C thereafter [2]. The indicating silica gel in each vial remained dry throughout the storage period. Seed moisture contents were not determined in this study due to the insufficient seed number, but previous studies of these seed lots (after 39 years of storage) indicated ~6–7% eRH (at 20 °C) and moisture contents of 3% or lower [2,3]. Seeds of 12 species sampled from RBG Kew had been equilibrated at 15% RH and 15 °C prior to storage at −20 °C for periods of 8–35 years.

### 4.2. Germination Assays

Germination of UPM seeds was assessed on water-moistened, double-layered filter papers placed in 7 cm glass Petri dishes, whereas germination of the RBG Kew seed collections was assessed on 1% agar–water following 24 h soaking in water. Seeds were germinated in three replicates at the beginning of the experiment (control) and in two replicates after differential scanning calorimetry (DSC) analysis. The number of seeds per replicate ranged from 6 to 138, depending on the availability of material. Optimum germination conditions were chosen based on an earlier study on the same species and accessions from the UPM seed bank [2]. Filter papers were kept moist by wetting them regularly with distilled water, as required. Specific germination conditions were applied for each species, with alternating (25/10 °C; 25/15 °C) or constant (20 °C) temperature, followed by either 24 or 48 h soaking in 1 g/L gibberellic acid (GA_3_), or on GA_3_ in 1% agar–water, with the seed coat kept intact or removed. Seed was incubated in a growth chamber, with a 16 h light and 8 h dark photoperiod provided by cool white fluorescent tubes with light intensity of 30–50 µmol m^−2^ s^−1^. The criterion for germination was 1 mm radicle protrusion, and it was quantified regularly for 50 days or until all seed had germinated. A cut test was carried out at the end of the germination test to assess viability of non-germinated seed. Significant differences (95% confidence level) between germination proportions before and after the DSC analysis were assessed on the basis of a Z-test for proportions for independent groups of varying sample sizes [48].

### 4.3. Seed Lipid Thermal Analysis and Fatty Acid Compositions

Lipid thermal behavior of dry whole seeds was carried out on 17 species from UPM and four species from RBG Kew collection. A differential scanning calorimetry (DSC 7 Perkin-Elmer, Beaconsfield UK) was used, controlled by a Perkin Elmer TAC-7. The instrument was calibrated using indium, zinc, and pure water, as standard for cryogenic operations [49]. Two replicates of seed samples of 9–17 mg, depending on the species, were placed in preweighed aluminium pans, nonhermetically sealed, and weighed to record sample weight. Samples were cooled from +25 °C to −100 °C and then warmed to 30 °C at a cooling/warming rate of ±10 °C min^−1^. Thereafter, the seeds were removed from the pans and prepared for germination, as described above. To investigate the effects of slow cooling and annealing on the lipid thermal fingerprints, three species for which relatively high volumes of seed were available from the Kew bank (*Barbarea intermedia, B. napus* and *Erucastrum abyssinicum*) had two additional thermal analyses applied over the same temperature range: 1) cooling/warming rate of 2 °C min^−1^; 2) annealing at −5 °C for 1 h during cooling, within a cooling/warming regime of 2 °C min^−1^. The melting peak onset temperatures and the enthalpies for lipid transitions in this dry material were analyzed using the Pyris 7 software, in relation to the scanning baseline. Lipid melt enthalpies (ΔH) were calculated based on seed weight and are presented as mJ g^−1^. Seed fatty acid profiles were compiled using the Seed Oil Fatty Acids (SOFA) database (http://sofa.mri.bund.de/).

## 5. Conclusions

Multiple features of lipid thermal fingerprints obtained using DSC appear to have potential as biophysical markers of storage performance for seeds stored in facilities operating to international guidelines [50].

## Figures and Tables

**Table 1:** Post-storage total germination (radicle emergence) of 20 Brassicaceae species after 43–44 years in the Plant Germplasm Bank—Universidad Politécnica de Madrid (UPM) seed bank at c. −5 °C and low moisture content (dry indicator silica gel) or after 12–35 years at the Millennium Seed Bank of the Royal Botanic Gardens (RBG) Kew at −20 °C after drying to 15% relative humidity. After storage (and differential scanning calorimetry [DSC]), seeds were germinated at 25/15 °C (8 h light/16 h dark photoperiod) following 24 h soaking in water, except *Matthiola incana* seeds, which were sown at 25 °C. Other modifications to this basic method are noted in the table.

	Years of Storage in UPM (or RBG Kew) Bank	Germination (%)				
		Before Storage at UPM *^1^	After Storage at UPM and Pre-DSC	After Storage at UPM and Post-DSC	Before Storage at RBG Kew	After Storage at RBG Kew
*Alliaria petiolata* Cavara & Grande.	NA (32)	NA	NA	NA	92 S	24
*Alyssoides utriculata* Medik.	43 (NA)	100	100 ^§^ NS	100 ^§^ NS		
*Alyssum saxatile* L.	43 (25)	100	100 NS	100 NS	100 NS	100
*Arabis turrita* L.	43 (NA)	0	4 ^‡^ NS	24 ^‡^ S		
*Barbarea intermedia* Boreau.	43 (NA)	95	100 NS	93 NS		
*Brassica napus* L.	43 (33)	100	100 NS	100 NS	100 NS	100
*Coincya rupestris* L.	43 (NA)	92	86 NS	100 S		
*Conringia orientalis* (L.) C.Presl.	44 (NA)	0	100 ^‡^ S	100 ^‡^ NS		
*Descurainia sophia* L.	44 (23)	2	100 ^‡^ S	96 ^‡^ NS	100 S	90
*Erucastrum abyssinicum* O.E.Schulz.	44 (NA)	100	90 S	98 NS		
*Erysimum cheiri* Crantz.	43 (34)	100	100 NS	100 NS	100 NS	100
*Erysimum odoratum* Baumg.	43 (NA)	100	100 NS	100 NS		
*Erysimum repandum L.*	43 (NA)	100	100 NS	100 NS		
*Lesquerella gordonii* Watson.	NA (8)	NA	NA	NA	96 S	0
*Matthiola incana* (L.) W.T.Aiton.	43 (35)	95	100 NS	100 NS	100 NS	100
*Matthiola sinuata* (L.) W.T.Aiton.	43 (22)	100	100 ^§^ NS	40 ^§^ S	100 NS	100
*Moricandia arvensis* (L.) DC.	44 (15)	20	96 ^†^ S	96 ^†^ NS	100 NS	94
*Rorippa palustris* (L.) Besser	NA (11)	NA	NA	NA	100 NS	100
*Sinapis arvensis Sinapis arvensis* L.	43 (12)	3	73 ^†^ S	85 † S	100 S	90
*Sisymbrium orientale* L.	43 (24)	3	100 ^‡^ S	99 ^‡^ NS	100 S	81

Z-test: germination data before and after storage and pre- and post-DSC are significantly (S) or not significantly (NS) different (*P* < 0.05); ^§^ 48 h presoak in water; ^†^ 24 h soak in 1 g/L gibberellic acid (GA_3_) solution; ^‡^ 48 h soak in 1 g/L GA_3_ solution, or on 1% agar with 1 g/L GA_3_ (for *Descurainia sophia* and *Sisymbrium orientale*); NA = not available; *^1^ [2]; 60 seeds sown at 23–25 °C with no pretreatments; UPM: Universidad Politécnica de Madrid. RBC: Royal Botanic Gardens. DSC: differential scanning calorimetry

**Table 2:** Lipid content, fatty acid composition, and thermal analysis during warming at 10 °C min^−1^ of dry seeds of 20 Brassicaceae species after 43–44 years in the Plant Germplasm Bank—Universidad Politécnica de Madrid (UPM) seed bank at c. −5 °C and low moisture content (ultradry with silica gel) or after 12–35 years at the Millennium Seed Bank of the Royal Botanic Gardens (RBG) Kew at −20 °C after drying to 15% RH. Values are the average of two replicates (± SEM), based on 6 to 138 seeds analyzed from the conservation collections and published literature. Fatty acid composition data was compiled from the Seed Oil Fatty Acids (SOFA) database (http://sofa.mri.bund.de/).

Species	Lipid Content (%)	Melt Onset T (°C)	Peak Mid-point (°C)	Melt End T (°C)	Melt Enthalpy (Δ*H*) (mJ g^−1^DW)	16:0 (Pal-mitic Acid)	18:0 (Stearic Acid)	18:1 (Oleic Acid)	18:2 (Linoleic Acid)	18:3 (Lino-lenic Acid)	20:1 (Gondoic Acid)	21:0 (HeneiCosylic Acid)	22:1 (Erucic Acid)	24:1 (Nervonic Acid)
*Alliaria petiolata*	28.0 (± 2.6) ^5,7^	−2.1 (± 0.7)	12.7 (± 0.1)	17.5 (± 0.8)	18.41 (± 0.2)	3.5	7.0	9.0	24.8	7.2	5.3	-	44.2	6.1
*Alyssoides utriculata*	12.7 (± 0.5) ^2,7^	−49.8 (± 0.0)	−42.7 (± 0.6)	−36.4 (± 0.8)	2.33 (± 0.6)	7	3	17	11	71.1	0.6	-	2.7	0.4
*Alyssum saxatile*	18.2 (± 4.7) ^2,7^	−50.9 (± 0.2)	−44.9 (± 0.1)	−35.3 (± 0.8)	5.98 (± 0.1)	5	1	12	20	58	0	-	0	0
*Arabis turrita*	NA	−50.3 (± 0.1)	−45.3 (± 0.0)	−40.3 (± 0.2)	3.22 (± 0.8)	NA	NA	NA	NA	NA	NA	NA	NA	NA
*Barbarea intermedia*	30.0 ^3^	−14.4 (± 0.2)	−5.1 (± 0.3)	−2.3 (± 0.2)	19.37 (± 0.3)	NA	NA	NA	NA	NA	NA	NA	NA	NA
*Brassica napus*	40.5 (± 2.3) ^4,7^	−10.1 (± 0.4) [−12.9] * (± 0.2)	−2.1 (± 0.1) [−4.2] * (± 0.1)	1.7 (0) [−1.7] * (± 0.1)	30.92 (± 0.8) [31.12] * (± 0.4)	3	0.9	14	15	8	10	-	55	0
*Coincya rupestris*	17.45 ^7^	−41.1 (± 0.8)	−33.2 (± 0.3)	−21.7 (± 0.0)	7.63 (± 0.2)	3	1.5	13.5	16.3	25.7	6.1	0.5	30.5	3.5
*Conringia orientalis*	26.5 (± 1.1) ^4,7^	−15.0 (± 0.0)	−8.3 (± 0.2)	−5.9 (± 0.1)	20.92 (± 0.2)	2.9	0.3	7	33	3.5	24.2	-	25.4	3.5
*Descurainia sophia*	37.1 (± 2.3) ^4,7^	−49.6 (± 1.2)	−36.7 (± 0.4)	−27.3 (± 0.4)	7.79 (± 0.8)	6	2	14	17	37	12.5	-	9	0
*Erucastrum abyssinicum*	34.0 ^5^	−12.4 (± 0.0)	−4.7 (± 0.1)	−2.1 (± 0.1)	21.04 (± 1.2)	3	1	10	18	13	8	-	40	2
*Erysimum cheiri*	29.5 ^4^	−42.7 (± 0.5)	−34.1 (± 0.2)	−27.4 (± 1.2)	5.97 (± 0.8)	NA	NA	NA	NA	NA	NA	NA	NA	NA
*Erysimum odoratum*	30.5 (± 0.8) ^2,7^	−45.6 (± 0.1)	−33.5 (± 0.2)	−24.6 (± 0.4)	7.86 (± 0.3)	3.1	0.8	3.6	18	30.2	5.4	-	28.6	3.6
*Erysimum repandum*	26.7 (± 6.3) ^2,7^	−46.9 (± 0.8)	−35.8 (± 0.5)	−23.4 (± 2.4)	6.45 (± 0.4)	5.9	1.7	9.3	17	33.0	2	-	17	-
*Lesquerella gordonii*	27.3 (± 0.1) ^5,7^	−5.6 (± 0.2)	12.2 (± 0.3)	17.4 (± 0.1)	5.85 (± 0.1)	2	2	22	4	7	1.0	57.9	-	-
*Matthiola incana*	20.8 ^2^	−44.3 (± 0.5)	−38.0 (± 1.0)	−31.7 (± 1.4)	2.31 (± 0.2)	8	2.5	13.6	9	62	0.5	-	0.8	0
*Matthiola sinuata*	29.0 ^5^	−47.0 (± 0.3)	−41.2 (± 0.1)	−34.2 (± 0.4)	9.46 (± 0.2)	NA	NA	NA	NA	NA	NA	NA	NA	NA
*Moricandia arvensis*	32.4 (± 0.9) ^6,7^	−40.7 (± 0.7)	-32.8 (± 0.3)	−27.5 (± 0.4)	3.95 (± 0.2)	10.4	1.7	11.5	19.6	32.6	5.3	-	20	0.5
*Rorippa palustris*	21.0 ^4^	−48.3 (± 1.2)	−38.6 (± 0.8)	−20.7 (± 0.7)	5.63 (± 0.4)	NA	NA	NA	NA	NA	NA	NA	NA	NA
*Sinapis arvensis*	28.4 (± 1.1) ^4,7^	−15.5 (± 1.0)	−6.3 (± 0.7)	−2.8 (± 0.4)	20.00 (± 0.8)	3.9	0.9	19.9	16	19.3	10.8	-	34.2	1.2
*Sisymbrium orientale*	22.1 (± 0.1) ^2,7^	−42.9 (± 0.2)	−34.8 (± 0.3)	−25.1 (± 0.4)	3.61 (± 0.2)	9.4	0.9	6.4	13.3	41.4	3.6	17.3	-	0.6

^2^ [34]; ^3^ [35]; ^4^ [32]; ^5^ [36]; ^6^ [29], ^7^ [37]. Values indicated in the brackets followed by * for *Brassica napus* show melt temperature for RBG Kew stored seeds.

## Supplementary Materials

The following are available online at https://www.mdpi.com/2223-7747/8/10/414/s1, Figure S1: Storage of Brassicaceae seeds in ultradry condition at UPM
