# Supplementary material for: Lipid Thermal Fingerprints of Long-term Stored Seeds of Brassicaceae
Source: Plants (Basel). 2019 Oct 14;8(10):414. doi: 10.3390/plants8100414 (PMC6843794; doi:10.3390/plants8100414)
Supplement: Supplementary file 1 [file plants-08-00414-s001.pdf]

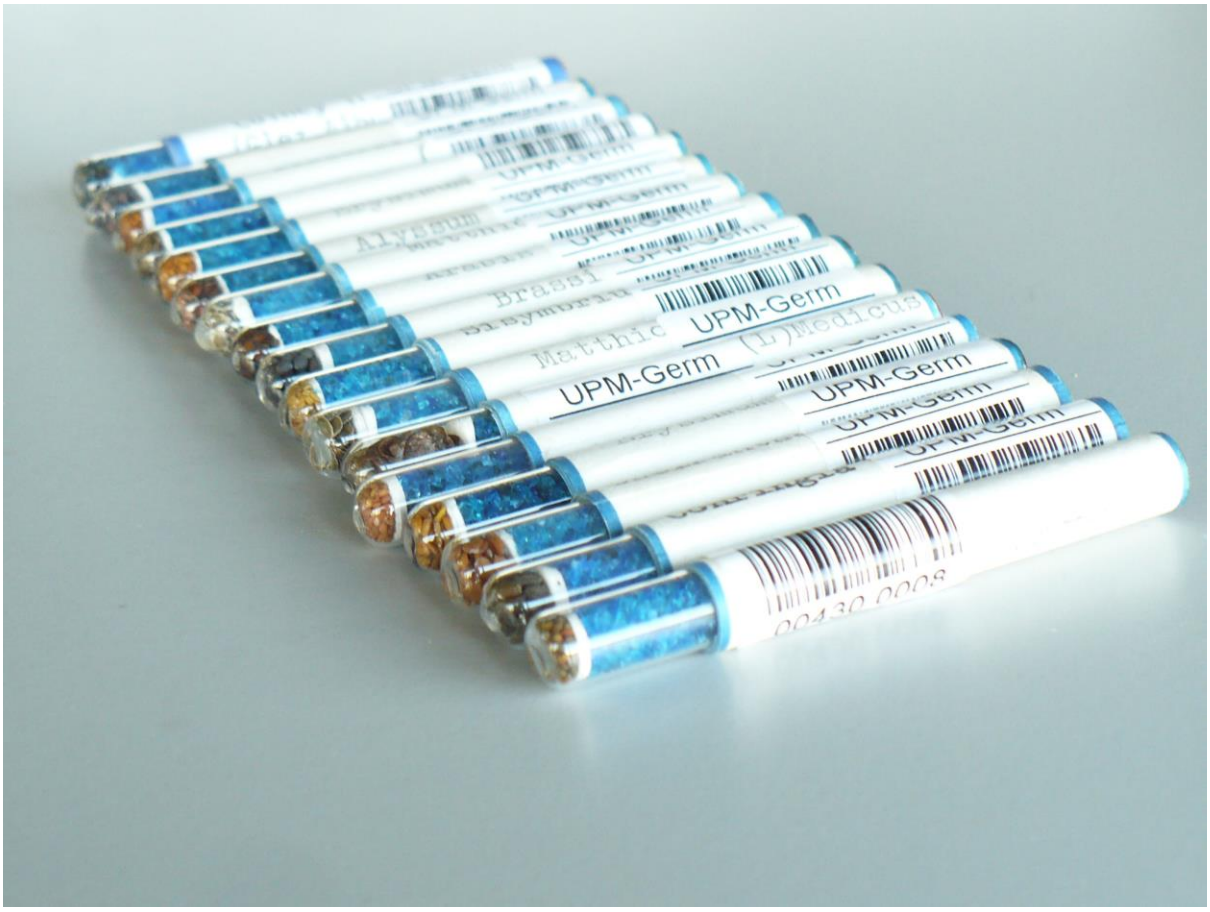

Figure S1. UPM Brassicaceae seed collections were stored in ultra-dry, cold conditions inside flame-sealed glass vials containing approximately 39 to 104 mg of dry seeds, together with silica gel, separated by a filter paper divider.
